# Excess body weight and specific types of depressive symptoms: Is there a mediating role of systemic low-grade inflammation?

**DOI:** 10.1016/j.bbi.2022.11.016

**Published:** 2023-02

**Authors:** Keqin Chu, Dorina Cadar, Eleonora Iob, Philipp Frank

**Affiliations:** a Department of Behavioural Science and Health, University College London, London, UK; b Brighton and Sussex Medical School, Brighton, East Sussex, UK; c Social, Genetic & Developmental Psychiatry (SGDP) Centre, Institute of Psychiatry, Psychology & Neuroscience, King’s College London, London, UK

**Keywords:** Excess body weight, Obesity, Overweight, Inflammation, Somatic depressive symptoms, Cognitive-affective depressive symptoms, C-reactive protein, Body mass index, Mediation

## Abstract

**Objectives:**

Obesity is associated with an increased risk of depression. Systemic low-grade inflammation, a plausible consequence of obesity, has also been linked to depression. However, the potential mediating effects of systemic low-grade inflammation on the association between excess body weight and specific symptom domains of depression remain uncertain. This study examined whether systemic low-grade inflammation mediated the associations of excess body weight (overweight and obesity) with subsequent overall, cognitive-affective, and somatic depressive symptoms.

**Design:**

This study used a prospective cohort design.

**Methods:**

The final analytical sample included 4,942 adults aged ≥50 years drawn from the English Longitudinal Study of Ageing (ELSA). Body mass index (BMI) and covariates were ascertained at baseline (wave 4, 2008/09). Continuous BMI scores were divided into four categories: ‘normal weight’ (18.5 ≤ BMI <25 kg/m^2^); ‘overweight’ (25 ≤ BMI <30 kg/m^2^); ‘obesity’ (BMI ≥30 kg/m^2^); in addition to ‘excess body weight’ (‘overweight’ and ‘obesity’ combined). Covariates included sociodemographic variables, behavioural factors, and chronic physical conditions. Serum concentrations of CRP were measured at wave 6 (2012/13). Depressive symptoms were assessed at baseline and ten years later (wave 9, 2018/19), using the 8-item Centre for Epidemiological Studies Depression (CES-D) Scale. Two symptom domains were constructed, distinguishing between cognitive-affective (depressed mood, loneliness, sadness, enjoyment in life, and happiness) and somatic (sleep problems, low energy levels, and fatigue) symptoms. Mediation analyses were performed to examine whether CRP statistically mediated the associations between BMI categories and depressive symptoms.

**Results:**

In multivariable-adjusted analyses, excess body weight was associated with elevated somatic (OR = 1.231, 95% CI: 1.029, 1.473), but not cognitive-affective or overall depressive symptoms at follow-up. Higher CRP was associated with elevated somatic (OR = 1.156, 95% CI: 1.061, 1.259), but not cognitive-affective or overall depressive symptoms. CRP acted as a partial mediator (14.92%) of the association between excess body weight and elevated somatic, but not cognitive-affective, or overall depressive symptoms.

**Conclusion:**

Systemic low-grade inflammation may partially explain the association of excess body weight with somatic depressive symptoms, but not the associations with cognitive-affective or overall depressive symptoms.

## Highlights

• Depression heterogeneity may lead to variability in its links with excess body weight.
• Excess body weight was associated with somatic but not cognitive-affective symptoms.
• CRP partially explained the link between excess body weight and somatic symptoms.

## 1 Introduction

As a leading cause of disability, depression poses a significant challenge to public health (Vos et al., 2017). To date, approximately 280 million people are affected by depression globally (World Health Organization, 2021a), and an estimated 18% of English adults aged 65 years and older experience elevated depressive symptoms (Zivin et al., 2010). Metabolic conditions such as obesity may contribute to the development of both subclinical depression and major depressive disorder in later life (Roberts et al., 2003, Vogelzangs et al., 2009). Over the past decades, global obesity rates have nearly tripled, with more than 1.9 billion adults classifying as overweight (25 ≤ body mass index (BMI) < 30 kg/m^2^) and over 650 million as obese (BMI ≥30 kg/m^2^) (World Health Organization, 2021b). Depression and excess body weight have been independently linked to an array of negative health outcomes, including impaired physical functioning (Penninx et al., 1999, Walter et al., 2009) and reduced quality of life (Andreyeva et al., 2007, Penninx et al., 2013). In addition, excess body weight, in particular obesity, has been associated with complex multimorbidity (*e.g.,* obesity-related cardiovascular, endocrine, digestive, genitourinary, skin, and blood diseases) (Kivimäki et al., 2022) and premature death (van Dam et al., 2006). While overweight and obesity are important conditions in their own right, evidence suggests that they may also contribute to poorer treatment responses to existing antidepressant therapies (Kloiber et al., 2007). With a progressively ageing population, exploring biological mechanisms underlying the association of excess body weight with depression in older adults has become a research priority.

Depression is a heterogeneous mental disorder characterised by varying types of symptom expressions (Jentsch et al., 2015). Symptoms of depression can be broadly categorised into somatic (*e.g.,* changes in appetite, fatigue, and sleep disturbance) and cognitive-affective symptoms (*e.g.,* depressed mood, and suicidal ideation) (American Psychiatric Association, 2013, Frank et al., 2020, Iob et al., 2020). It has been hypothesised that depression heterogeneity may contribute to variability in the association between excess body weight and depression (Milaneschi et al., 2019). Consistent with this notion, there is some evidence of a stronger association between excess weight and somatic symptoms *(e.g.,* altered appetite and sleep problems), whereas the association with cognitive-affective symptoms (*e.g.,* unhappiness and loss of interest) appears to be weaker (Frank et al., 2021, Wiltink et al., 2013).

One proposed biological mechanism by which obesity may contribute to depression aetiology is systemic low-grade inflammation (Capuron et al., 2017). Systemic low-grade inflammation refers to the continuous production of pro-inflammatory markers in response to immune challenges (Pietzner et al., 2017). According to the inflammation hypothesis of depression (Alexopoulos and Morimoto, 2011), ageing may stimulate aberrant immune responses in the peripheral nervous system (PNS) and impair PNS-CNS (central nervous system) immune communication. These immune-related changes may then induce a series of depressive-like symptoms, also known as ‘sickness behaviour’ *(e.g.,* low mood, memory deficits, sleep problems, and social withdrawal) (Dantzer et al., 2008). Common markers of systemic inflammation include cytokines, such as interleukin-6 (IL-6) and the acute phase reactant C-reactive protein (CRP) (Smith et al., 2018). Previous evidence shows that higher CRP and IL-6 levels are associated with future depressive symptoms in older adults (Zalli et al., 2016), suggesting potential depressogenic effects of systemic low-grade inflammation.

While some longitudinal investigations have reported positive associations between CRP and subsequent depressive symptoms (Niles et al., 2018, van den Biggelaar et al., 2007), others have yielded mixed or null results (Au et al., 2015, Stewart et al., 2009). For example, a recent meta-analytic synthesis of longitudinal data (Mac Giollabhui et al., 2021) found that only four of twelve studies on older adults (≥60 years) reported positive associations between CRP and subsequent depressive symptoms (Das, 2017, Niles et al., 2018, van den Biggelaar et al., 2007, Zalli et al., 2016). It has been hypothesised that inconsistencies in findings may be attributable to heterogeneity in symptom profiles, in addition to methodological differences (Mac Giollabhui et al., 2021). Notably, recent evidence suggests that CRP may be primarily associated with somatic rather than cognitive-affective symptoms (Frank et al., 2021, Majd et al., 2020).

Systemic low-grade inflammation is also a hallmark of adulthood obesity (Kanneganti and Dixit, 2012). Adipose tissue represents a key determinant for low-grade inflammation (Yudkin et al., 1999). Specifically, macrophage infiltration into obese adipose tissue can induce the secretion of inflammatory factors such as IL-6 (Xu et al., 2003), followed by the synthesis of CRP in the liver (Shoelson et al., 2007). Accordingly, evidence suggests that macrophage markers in visceral adipose tissue are associated with systemic inflammation in patients with obesity (Lasselin et al., 2014). Furthermore, obesity-related inflammation has been found to increase the risk of developing comorbid depression via, for example, alterations in hypothalamic–pituitary–adrenal (HPA) axis function, monoamine metabolism, and neurocircuitry involving primarily the basal ganglia and the subgenual anterior cingulate cortex (Capuron and Miller, 2011, Castanon et al., 2014). A reduction of inflammatory markers after weight loss has also been shown to alleviate depressive symptomatology in individuals with obesity (Emery et al., 2007). Altogether, these findings suggest that systemic low-grade inflammation may act as a potential mediator of the relationship between excess body weight, in particular obesity, and depressive symptoms (Capuron et al., 2017). To date, however, relatively few studies have investigated the mediating role of systemic inflammation in the association between excess body weight and subsequent depressive symptoms in older adults. A previous prospective cohort study of 3,891 English community-dwelling older adults showed that higher CRP represented a partial mediator of the relationship between obesity and subsequent overall depressive symptoms (Daly, 2013). However, since both CRP and obesity in this study were assessed at the same point in time, temporality could not be inferred, and domain-specific associations were not examined.

Hence, the present study aimed to investigate the associations of excess weight (*i.e.,* overweight, obesity, overweight + obesity) with overall depressive symptoms and symptom-specific domains (*i.e.,* cognitive-affective and somatic symptoms) in a community-based sample of English older adults, using prospective data from the English Longitudinal Study of Ageing. We also examined whether higher concentrations of CRP had a mediating role in the associations of excess body weight with subsequent overall, cognitive-affective, and somatic depressive symptoms. It was hypothesised that:

1. Higher baseline BMI levels (overweight, obesity, overweight + obesity) would be associated with elevated overall, cognitive-affective, and somatic depressive symptoms at follow-up.
2. Higher baseline BMI levels (overweight, obesity, overweight + obesity)  and CRP would be more strongly associated with elevated somatic rather than cognitive-affective depressive symptoms at follow-up.
3. Higher CRP levels would statistically mediate the associations of higher baseline BMI levels (overweight, obesity, overweight + obesity)  with elevated overall, cognitive-affective, and somatic depressive symptoms.

## 2 Methods

### 2.1 Study design

A prospective cohort design was adopted, based on data from the English Longitudinal Study of Ageing (ELSA) – a nationally representative, observational study of men and women aged ≥50 years living in England (Steptoe et al., 2013). Launched in 2002, ELSA has collected a variety of information on the ageing process, including health-related, psychosocial, and economic data. The original sample was derived from households participating in the Health Survey for England (HSE) in 1998, 1999, and 2001 (Mindell et al., 2012). Since study inception, participants have been followed up biannually. Psychosocial data were collected via face-to-face computer-assisted interviews (CAPI) and self-report questionnaires. Biological data were gathered quadrennially by trained nurses. In the present study, baseline data on BMI (exposure) and covariates were collected at wave 4 (2008/09). Depressive symptoms (outcome) were assessed at wave 9 (2018/19), the latest wave of data collection available during the time of the analysis. CRP (mediator) was measured four years after baseline (wave 6, 2012/13). Informed consent was obtained from all ELSA participants prior to their participation in the study. Ethical approval for all the ELSA waves was granted by the National Research and Ethics Committee.

### 2.2 Participants

**Fig. 1:**
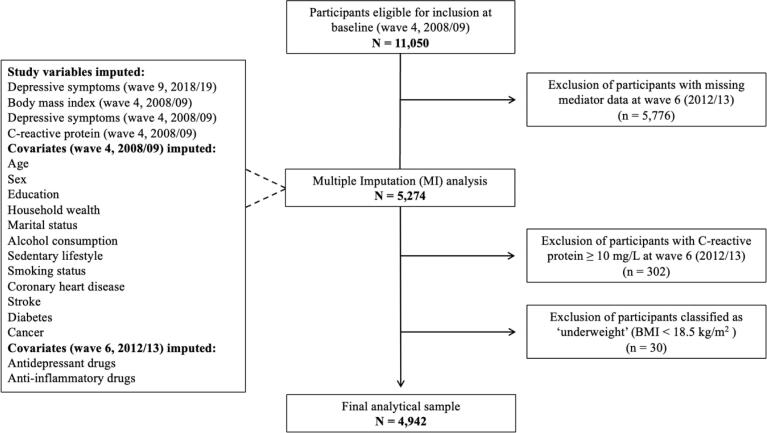
Participant flowchart for the analytical sample.

A total of 11,050 participants were eligible for inclusion at wave 4. Participants who did not provide blood samples at wave 6 (n = 5,776) were excluded from the present analysis (Collins et al., 2001). Individuals with CRP ≥ 10 mg/L at wave 6 (n = 302) were omitted because values above this threshold may denote acute infection rather than systemic low-grade inflammation (Myers et al., 2004). Respondents with BMIs < 18.5 kg/m^2^ (*i.e.,* underweight) were also excluded from the analytical sample (n = 30) since there were too few cases to conduct sufficiently powered analyses. The final analytical sample consisted of 4,942 people (see Fig. 1 for the participant flowchart).

### 2.3 Measures

#### 2.3.1 Exposure: BMI categories at wave 4 (2008/09)

BMI was computed from weight in kilograms divided by height in metres squared (kg/m^2^). Participants’ weight was measured using a portable electronic scale, and their height via a portable stadiometer. BMI values were classified into four categories: ‘normal weight’ (18.5 ≤ BMI < 25 kg/m^2^); ‘overweight’ (25 ≤ BMI < 30 kg/m^2^); ‘obesity’ (BMI ≥ 30 kg/m^2^) (World Health Organization, 2010); in addition to a combined weight group ‘excess body weight’ (‘overweight’ + ‘obesity’).

#### 2.3.2 Outcome: Depressive symptoms at wave 9 (2018/19)

Depressive symptoms were ascertained from the 8-item Centre for Epidemiological Studies Depression (CES-D) Scale (Radloff, 1977). The CES-D has shown adequate internal consistency and similar psychometric properties to the full 20-item scale (Turvey et al., 1999). Participants responded to eight items on a dichotomous scale (yes = 1; no = 0), indicating whether in the last two weeks they had experienced a) depressed mood, b) loneliness, c) sadness, d) enjoyment in life, e) happiness, f) lower energy levels, g) restless sleep, and h) effort doing things. Positively phrased items (enjoyment in life, happiness) were reverse coded. Sum scores ranged from 0 to 8, and a validated cut-off score of ≥ 4 was used to denote ‘elevated overall depressive symptoms’ (Steffick, 2000). Two additional sum scores were computed from a predefined set of individual CES-D items to assess cognitive-affective [items a, b, c, d, and e] and somatic [items f, g, and h] symptom domains. Domain-specific sum scores were further dichotomised, using the upper tertile as an indicator of ‘high symptom load’, respectively (CES-D ≥ 2 for cognitive-affective symptoms and CES-D ≥ 2 for somatic symptoms). In the present sample, the internal consistency coefficient (Cronbach’s alpha) was 0.78 for the 8-item CES-D, 0.77 for the ‘cognitive-affective’ domain, and 0.57 for the ‘somatic’ domain (Cronbach, 1951).

#### 2.3.3 Mediator: C-reactive protein at wave 6 (2012/13)

Non-fasting blood (6 mL) was drawn by a nurse via antecubital venepuncture. Blood samples were centrifuged, and serum was stored at −70 °C until analysis. Serum concentrations of CRP (mg/L) were assessed with the N Latex CRP mono Immunoassay on the Behring Nephelometer II Analyser, at the Royal Victoria Infirmary laboratory in Newcastle (United Kingdom). The detection limit was 0.17 mg/L, and the coefficient of variation (CV) was less than 6%. Further details can be found in the 2004 HSE technical report (Graig et al., 2006). CRP concentrations were log-transformed due to their skewed distribution.

#### 2.3.4 Baseline covariates

Covariates captured at baseline included sociodemographic, behavioural, and illness-related factors. Sociodemographic variables were age, sex, marital status, education, and wealth. Marital status was divided into two groups (single and married). Education was categorised into three groups (university degree or above, lower than a university degree, and no qualification). Wealth was ascertained from participants’ total household wealth, which comprised information on participants’ property wealth, financial wealth, physical wealth, and business assets, minus debts. In ELSA, this variable is provided in quintiles, with participants in the highest quintile classified as the wealthiest group.

Behavioural factors included smoking status (smoker or non-smoker), frequency of alcohol consumed in the last twelve months (daily or less than daily), and sedentary lifestyle (yes or no). Data on sedentary lifestyle were obtained by asking participants whether they had engaged in mild/moderate/vigorous exercise at least once per week. Responses were categorised as ‘yes’ (no exercise) versus ‘no’ (mild/moderate/vigorous exercise at least once per week).

Chronic conditions were self-reported doctor-diagnosed indications of coronary heart disease, stroke, diabetes, and cancer.

### 2.4 Statistical analyses

Baseline characteristics of the study participants are reported as means and standard deviations (SD) for continuous variables, and proportions for categorical variables. Variables that were skewed (CRP) are presented as geometric means and interquartile ranges. Differences in means and proportions of covariates between included and excluded participants were tested using two-sided t-tests and Chi-square tests, respectively. Differences in CRP levels by weight (normal weight, overweight, and obesity) and depression status (low versus elevated depressive symptoms) were examined using a one-way ANOVA and two-sided t-tests, respectively.

**Fig. 2:**
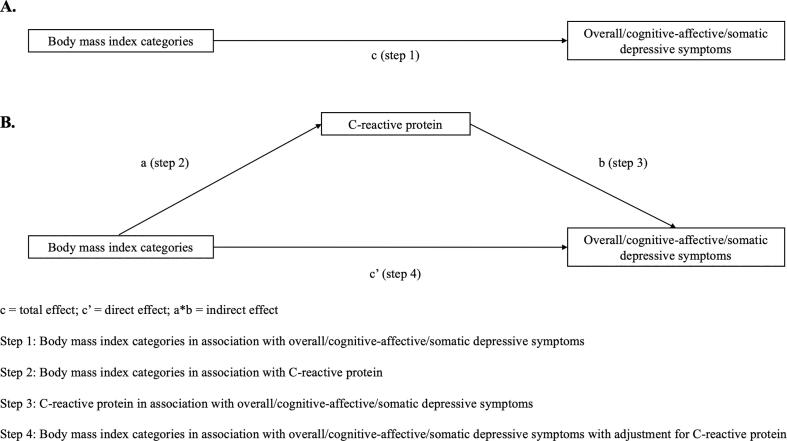
Hypothesised mediation model.

A series of multivariate regression analyses were performed to assess whether the criteria for conducting mediation analysis were met (see Fig. 2 for graphical illustrations). First, hierarchical logistic regression analyses were carried out to examine the associations of baseline BMI categories with subsequent overall, cognitive-affective, and somatic depressive symptoms (step 1, Fig. 2A), as well as between CRP (wave 6) and subsequent overall, cognitive-affective, and somatic depressive symptoms (step 3, Fig. 2B). Second, hierarchical linear regressions were conducted to test the associations of baseline BMI categories with CRP (step 2, Fig. 2B). Potential confounding factors were entered in a stepwise fashion: Model 1 – age, sex, and baseline depressive symptoms (overall depressive symptoms, cognitive-affective symptoms, or somatic symptoms, depending on the outcome of interest; steps 1 and 3) or CRP (step 2); Model 2 – Model 1 and sociodemographic variables; Model 3 – Model 2 and behavioural factors; and Model 4 – Model 3 and chronic conditions. The results are presented as odds ratios (ORs) with 95% accompanying confidence intervals (CI) for logistic regression analyses, and unstandardised coefficients B with 95% CI for linear regressions.

Mediation analyses were performed using the ‘medeff’ statistical package in Stata (Hicks and Tingley, 2011). The total effect describes the effect of BMI categories on overall, cognitive-affective, and somatic depressive symptoms, respectively (path c, Fig. 2A). The indirect effect refers to the amount of mediation exerted on the relationship between BMI categories and depressive symptoms via CRP (path a*b, Fig. 2B). The direct effect represents the effect of BMI categories on depressive symptoms with adjustment for CRP (path c’, Fig. 2B). Because the ‘medeff’ package only allows for binary exposure variables, three dummy variables [(1) ‘excess body weight (denoted by the combined weight group: overweight + obesity)’ (n = 4,942), (2) ‘overweight’ (n = 3,507), and (3) ‘obesity’ (n = 2,753)] were created, with ‘normal weight’ representing the reference category. The analysis comparing ‘excess body weight’ with normal weight was considered the primary mediation analysis as these analyses maintained the original sample size and were therefore of greater statistical power. All analyses were performed using Stata/SE version 17.

Missing values of the exposure, mediator, outcome and confounding variables were estimated using multiple imputation (MI) analysis (Rubin, 1987). Five imputed datasets with 20 iterations were performed, and effect estimates from both the regression and mediation models were pooled according to Rubin’s rules (Rubin, 1987). We performed a post-hoc power-analysis using G*Power 3.1 (Faul et al., 2009), which revealed a minimum sample size of 954 (see supplementary material for more details).

### 2.5 Sensitivity analyses

Four sensitivity analyses were conducted. The first tested whether associations observed in the main analyses were attributable to treating the depression scores as categorical rather than continuous variables. Hence, linear regression analyses were conducted to investigate the associations between BMI categories and continuous depressive symptom scores (Supplementary Table S4). Second, mediation analyses were repeated with additional adjustment for antidepressant and anti-inflammatory drugs at wave 6 to determine whether associations occurred independently of drug use (Supplementary Table S5). Third, we repeated mediation analyses using alternative dummy variables as exposures [(1) ‘obesity versus normal weight + overweight’ (n = 4,942) and (2) ‘overweight versus normal weight + obesity’ (n = 4,942)] to test whether the categorisation of BMI scores in our main analyses may have resulted in a loss of power (Supplementary Table S6). Lastly, regression analyses stratified by sex were performed to evaluate whether relationships between BMI categories and subsequent depressive symptoms differed between men and women (Supplementary Table S7).

## 3 Results

### 3.1 Sample characteristics

Baseline characteristics of the study participants are provided in Table 1. Sample characteristics before and after multiple imputations are reported in Supplementary Table S1. The average age was 64.00 ± 8.51 years in the analytic sample (54.84% women). Approximately 44% of the participants were overweight, and 29% were obese at baseline. At follow-up, elevated depressive symptoms (CES-D ≥ 4) were found in 13% of participants, whereas 21% reported elevated somatic, and 15% reported elevated cognitive-affective depressive symptoms (upper tertile). The geometric mean concentration of CRP at wave 6 was 1.46 mg/L, with an interquartile range from 0.8 to 2.8. Relative to participants with normal weight, those with obesity were more likely to have higher levels of CRP. A similar trend was observed in participants with elevated versus non-elevated depressive symptoms (Supplementary Table S2). A total of 549 (38.26%) participants with obesity had CRP levels ranging between 3 and 10 mg/L (Supplementary Table S2).

**Table 1:** Descriptive characteristics of the analytic sample.

Analytic sample (N = 4,942)	Mean (SD)/Nr. (%)
Elevated overall depressive symptoms (CES-D ≥ 4)	
wave 9	636 (12.87%)
wave 4	577 (11.68%)

Elevated cognitive-affective symptoms (upper tertile: CES-D ≥ 2)	
wave 9	754 (15.26%)
wave 4	720 (14.57%)

Elevated somatic symptoms (upper tertile: CES-D ≥ 2)	
wave 9	1,059 (21.43%)
wave 4	782 (15.82%)

BMI at wave 4 (kg/m^2^)	
*Normal weight (18.5 ≤ BMI < 25)*	1,318 (26.67%)
*Overweight (25 ≤ BMI < 30)*	2,189 (44.29%)
*Obesity (BMI ≥ 30)*	1,435 (29.04%)
*Excess body weight (‘overweight’ and ‘obesity’ combined)*	3,624 (73.33%)

C-reactive protein (mg/L)*	
wave 6	1.46 (0.8, 2.8)
wave 4	0.84 (0.8, 3.6)

Age	64.00 ± 8.51

Sex	
*Men*	2,232 (45.16%)
*Women*	2,710 (54.84%)

Education	
*University degree*	1,900 (38.45%)
*Less than university*	1,957 (39.60%)
*No qualification*	1,085 (21.95%)

Wealth (quintiles)*	
*Lowest quintile*	634 (12.83%)
*2nd quintile*	903 (18.27%)
*3rd quintile*	1,017 (20.58%)
*4th quintile*	1,108 (22.42%)
*Highest quintile*	1,280 (25.90%)

Marital status	
*Single*	1,445 (29.24%)
*Married*	3,497 (70.76%)

Smoking status	
*Smoker*	597 (12.08%)
*Non-smoker*	4,345 (87.92%)

Alcohol consumption	
*Less than daily*	3,788 (76.65%)
*Daily (5/7 per week)*	1,154 (23.35%)

Sedentary lifestyle	
*Yes*	535 (10.83%)
*No*	4,407 (89.17%)

Coronary heart disease ever	
*Yes*	302 (6.11%)
*No*	4,640 (93.89%)

Stroke ever	
*Yes*	128 (2.59%)
*No*	4,814 (97.41%)

Diabetes ever	
*Yes*	362 (7.32%)
*No*	4,580 (92.68%)

Cancer ever	
*Yes*	349 (7.06%)
*No*	4,593 (92.94%)

Antidepressant drugs at wave 6	
*Yes*	498 (10.08%)
*No*	4,444 (89.92%)

Anti-inflammatory drugs at wave 6	
*Yes*	373 (7.55%)
*No*	4,569 (92.45%)

*Note:* Mean (standard deviation) (95% confidence intervals), and Number (percentage).

Abbreviations BMI, body mass index CES-D, 8-item Centre for Epidemiological Studies Depression.

* C-reactive protein was based on the geometric mean and interquartile ranges.

* The cut-off points for the wealth group definition were: Lowest – less than £60 k; 2nd –between £60 k-£201 k; 3rd – between £201 k-£303 k; 4th – between £303 k-£496 k; Highest – more than £496 k.

Relative to included participants, those who were excluded from the present analysis due to missing data or CRP levels ≥ 10 mg/L were more likely to be obese (34.33% vs 29.04%, p < 0.001) and current smokers (15.58% vs 12.08%, p < 0.001). Furthermore, they were less likely to have an educational qualification (67.43% vs 78.05%, p < 0.001), drink alcohol (22.56% vs 23.35%, p < 0.001), and to belong to the highest quintile of household wealth (17.85% vs 25.90%, p < 0.001) (Supplementary Table S3).

### 3.2 Step 1: BMI categories and overall/cognitive-affective/somatic depressive symptoms

Table 2 shows the results of the hierarchical logistic regression models examining associations of baseline BMI categories with depressive symptoms at a ten-year follow-up. In the minimally adjusted model (Model 1: ORs adjusted for age, sex, and baseline depressive symptoms), overweight (versus normal weight) was not associated with elevated overall (Model 1: OR = 0.999, 95% CI: 0.795, 1.256), cognitive-affective (Model 1: OR = 0.932, 95% CI: 0.758, 1.146), or somatic (Model 1: OR = 1.051, 95% CI: 0.869, 1.271) symptoms. In contrast, obesity (versus normal weight) was associated with elevated overall (Model 1: OR = 1.378, 95% CI: 1.090, 1.743), cognitive-affective (Model 1: OR = 1.250, 95% CI: 1.009, 1.549), and somatic (Model 1: OR = 1.640, 95% CI: 1.348, 1.996) symptoms at follow-up. These associations were attenuated after multivariable adjustment (ORs additionally adjusted for socioeconomic status, lifestyle factors, and reported illnesses) and remained statistically signficant only in the analysis of the somatic symptom domain (Model 4: OR = 1.511, 95% CI: 1.229, 1.858). When combining overweight and obesity into a single BMI category, excess body weight (versus normal weight) was associated with somatic (Model 4: OR = 1.231, 95% CI: 1.029, 1.473), but not cognitive-affective (Model 4: OR = 1.009, 95% CI: 0.831, 1.223) or overall (Model 4: OR = 1.101, 95% CI: 0.889, 1.364) symptoms in multivariable-adjusted analyses.

**Table 2:** Associations of baseline excess body weight, overweight, and obesity (versus normal weight) (wave 4, 2008/09) with overall, cognitive-affective, and somatic depressive symptoms at follow-up (wave 9, 2018/19).

Model	Elevated overall symptoms (CES-D ≥ 4)		Elevated cognitive-affective symptoms (upper tertile)		Elevated somatic symptoms (upper tertile)	
	OR (95% CI)	*P-value*	OR (95% CI)	*P-value*	OR (95% CI)	*P-value*
Excess body weight*						
(N = 4,942)						
Model 1	1.153 (0.937, 1.418)	*0.178*	1.060 (0.879, 1.277)	*0.543*	1.275 (1.073, 1.516)	*0.006*
Model 2	1.076 (0.872, 1.329)	*0.495*	0.985 (0.815, 1.192)	*0.878*	1.224 (1.026, 1.460)	*0.025*
Model 3	1.106 (0.893, 1.369)	*0.356*	1.014 (0.837, 1.230)	*0.885*	1.230 (1.028, 1.471)	*0.023*
Model 4	1.101 (0.889, 1.364)	*0.379*	1.009 (0.831, 1.223)	*0.931*	1.231 (1.029, 1.473)	*0.023*

*Overweight*						
(N = 3,507)						
Model 1	0.999 (0.795, 1.256)	*0.996*	0.932 (0.758, 1.146)	*0.504*	1.051 (0.869, 1.271)	*0.611*
Model 2	0.979 (0.777, 1.233)	*0.855*	0.905 (0.734, 1.115)	*0.348*	1.046 (0.862, 1.268)	*0.648*
Model 3	1.012 (0.802, 1.277)	*0.921*	0.935 (0.757, 1.154)	*0.530*	1.067 (0.878, 1.296)	*0.516*
Model 4	1.011 (0.800, 1.277)	*0.927*	0.932 (0.755, 1.151)	*0.515*	1.067 (0.878, 1.297)	*0.512*

*Obesity*						
(N = 2,753)						
Model 1	1.378 (1.090, 1.743)	*0.007*	1.250 (1.009, 1.549)	*0.041*	1.640 (1.348, 1.996)	*<0.001*
Model 2	1.219 (0.957, 1.551)	*0.108*	1.105 (0.887, 1.377)	*0.373*	1.513 (1.237, 1.851)	*<0.001*
Model 3	1.249 (0.978, 1.597)	*0.075*	1.138 (0.910, 1.423)	*0.257*	1.501 (1.223, 1.843)	*<0.001*
Model 4	1.241 (0.969, 1.589)	*0.087*	1.129 (0.902, 1.414)	*0.289*	1.511 (1.229, 1.858)	*<0.001*

*Note:* Model 1: effect estimates adjusted for age, sex, and baseline depressive symptoms. Model 2: model 1 + education, wealth, and marital status. Model 3: model 2 + smoking status, alcohol consumption, and sedentary lifestyle. Model 4: model 3 + coronary heart disease, stroke, diabetes, and cancer.

Abbreviations: CES-D, 8-item Centre for Epidemiological Studies Depression; CI, confidence intervals; OR, odds ratio.

* Excess body weight was denoted by combining participants in the ‘overweight’ and ‘obesity’ categories.

### 3.3 Step 2: BMI categories and C-reactive protein

The results of the hierarchical linear regression models for the associations between baseline BMI categories and CRP at wave 6 are summarised in Table 3. Compared with normal weight, overweight (Model 4: B = 0.143, SE = 0.027, 95% CI: 0.089, 0.196), obesity (Model 4: B = 0.318, SE = 0.031, 95% CI: 0.256, 0.379), and excess body weight (denoted by the combined weight group: overweight + obesity, Model 4: B = 0.201, SE = 0.026, 95% CI: 0.150, 0.252) were associated with higher CRP, even after controlling for baseline CRP, sociodemographic variables, behavioural factors, and chronic conditions.

**Table 3:** Associations of baseline excess body weight, overweight, and obesity (versus normal weight) (wave 4, 2008/09) with C-reactive protein at wave 6 (2012/13).

Model	Log C-reactive protein			
	Unstandardised coefficients		t	*P-value*
	B (SE)	95% CI		
Excess body weight*				
(N = 4,942)				
Model 1	0.190 (0.026)	0.140, 0.240	7.41	*<0.001*
Model 2	0.186 (0.026)	0.136, 0.236	7.23	*<0.001*
Model 3	0.199 (0.026)	0.148, 0.249	7.66	*<0.001*
Model 4	0.201 (0.026)	0.150, 0.252	7.74	*<0.001*

*Overweight*				
(N = 3,507)				
Model 1	0.127 (0.027)	0.073, 0.180	4.64	*<0.001*
Model 2	0.127 (0.027)	0.074, 0.181	4.67	*<0.001*
Model 3	0.140 (0.027)	0.087, 0.194	5.13	*<0.001*
Model 4	0.143 (0.027)	0.089, 0.196	5.20	*<0.001*

*Obesity*				
(N = 2,753)				
Model 1	0.304 (0.031)	0.244, 0.364	9.91	*<0.001*
Model 2	0.294 (0.031)	0.233, 0.355	9.52	*<0.001*
Model 3	0.311 (0.031)	0.250, 0.372	9.95	*<0.001*
Model 4	0.318 (0.031)	0.256, 0.379	10.11	*<0.001*

*Note:* Model 1: effect estimates adjusted for age, sex, and baseline C-reactive protein. Model 2: model 1 + education, wealth, and marital status. Model 3: model 2 + smoking status, alcohol consumption, and sedentary lifestyle. Model 4: model 3 + coronary heart disease, stroke, diabetes, and cancer.

Abbreviations: B, regression coefficient; CI, confidence intervals; SE, Standard error.

* Excess body weight was denoted by combining participants in the ‘overweight’ and ‘obesity’ categories.

### 3.4 Step 3: C-reactive protein and overall/cognitive-affective/somatic depressive symptoms

The results of the hierarchical logistic regression models testing the associations between CRP at wave 6 and depressive symptoms at wave 9 are displayed in Table 4. Higher CRP was associated with subsequently elevated somatic symptoms (Model 4: OR = 1.156, 95% CI: 1.061, 1.259), and this relationship remained after multivariable adjustment (ORs adjusted for baseline depressive symptoms, sociodemographic variables, lifestyle factors, and chronic conditions). In contrast, no association was found between higher CRP and elevated overall (Model 4: OR = 1.047, 95% CI: 0.944, 1.161) or cognitive-affective (Model 4: OR = 0.985, 95% CI: 0.897, 1.082) symptoms at follow-up.

**Table 4:** Associations between C-reactive protein at wave 6 (2012/13) and overall, cognitive-affective, and somatic depressive symptoms at follow-up (wave 9, 2018/19) (N = 4,942).

Mediator	Elevated overall symptoms (CES-D ≥ 4)		Elevated cognitive-affective symptoms (upper tertile)		Elevated somatic symptoms (upper tertile)	
	OR (95% CI)	*P-value*	OR (95% CI)	*P-value*	OR (95% CI)	*P-value*
Log C-reactive protein						
Model 1	1.138 (1.031, 1.257)	*0.011*	1.063 (0.970, 1.164)	*0.190*	1.234 (1.135, 1.340)	*<0.001*
Model 2	1.066 (0.963, 1.181)	*0.220*	1.005 (0.915, 1.103)	*0.921*	1.171 (1.076, 1.275)	*<0.001*
Model 3	1.042 (0.940, 1.155)	*0.433*	0.982 (0.894, 1.079)	*0.712*	1.150 (1.055, 1.252)	*0.001*
Model 4	1.047 (0.944, 1.161)	*0.383*	0.985 (0.897, 1.082)	*0.756*	1.156 (1.061, 1.259)	*0.001*

*Note:* Model 1: effect estimates adjusted for age, sex, and baseline depressive symptoms. Model 2: model 1 + education, wealth, and marital status. Model 3: model 2 + smoking status, alcohol consumption, and sedentary lifestyle. Model 4: model 3 + coronary heart disease, stroke, diabetes, and cancer.

Abbreviations: CES-D, 8-item Centre for Epidemiological Studies Depression; CI, confidence intervals; OR, odds ratio.

### 3.5 Step 4: Mediation analyses

**Fig. 3:**
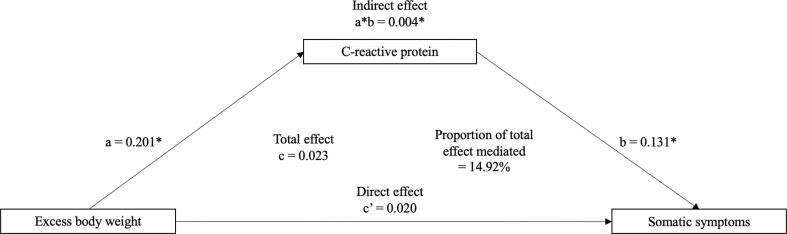
Mediation of the association between excess body weight (denoted by overweight + obesity) (versus normal weight) (wave 4, 2008/09) and subsequent somatic depressive symptoms (wave 9, 2018/19) via C-reactive protein (wave 6, 2012/13) (N = 4,942). Note. * = p < 0.05. Odds ratios were adjusted for sociodemographic variables (age, sex, education, wealth, and marital status), behavioural factors (smoking status, alcohol consumption, and sedentary lifestyle), chronic conditions (coronary heart disease, stroke, diabetes, and cancer), in addition to baseline C-reactive protein and depressive symptoms. A bias-corrected bootstrap using 1000 iterations was applied to all models.

**Fig. 4:**
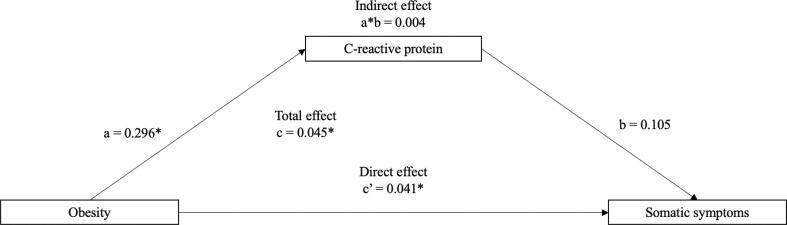
Mediation of the association between obesity (versus normal weight) (wave 4, 2008/09) and subsequent somatic depressive symptoms (wave 9, 2018/19) via C-reactive protein (wave 6, 2012/13) (N = 2,753). Note. * = p < 0.05. Odds ratios were adjusted for sociodemographic variables (age, sex, education, wealth, and marital status), behavioural factors (smoking status, alcohol consumption, and sedentary lifestyle), chronic conditions (coronary heart disease, stroke, diabetes, and cancer), in addition to baseline C-reactive protein and depressive symptoms. A bias-corrected bootstrap using 1000 iterations was applied to all models.

A total of two mediation analyses were performed to examine whether CRP statistically mediated the associations of (1) excess body weight (overweight + obesity, n = 4,942) and (2) obesity (n = 2,753) with subsequent somatic symptoms. No mediation analyses were conducted for overall or cognitive-affective symptoms because there was no association evident between BMI categories and these measures in prior analyses. Table 5, Fig. 3, and Fig. 4 show the results of the mediation analyses.

**Table 5:** Mediation of the associations of baseline excess body weight and obesity (versus normal weight) (wave 4, 2008/09) with somatic depressive symptoms at wave 9 (2018/19) through C-reactive protein at wave 6 (2012/13) (N = 4,942; N = 2,753)*.

Independent variable (wave 4)	Mediator (wave 6)	Outcome variable (wave 9)	Total indirect effect (a*b)	Total effect (c)	Total direct effect (c’)	Total effect mediated
			Coefficient (Bc CI)	Coefficient (Bc CI)	Coefficient (Bc CI)	%
Excess body weight*	CRP	Somatic symptoms	0.004 (0.001, 0.006)	0.023 (−0.000, 0.047)	0.020 (−0.004, 0.044)	14.92%

Obesity	CRP	Somatic symptoms	0.004 (−0.000, 0.010)	0.045 (0.016, 0.077)	0.041 (0.011, 0.073)	

*Note:* Effect estimates adjusted for sociodemographic variables (age, sex, education, wealth, and marital status), behavioural factors (smoking status, alcohol consumption, and sedentary lifestyle), chronic conditions (coronary heart disease, stroke, diabetes, and cancer), in addition to baseline C-reactive protein and depressive symptoms.

Abbreviations: Bc CI, Bias corrected 95% confidence intervals; CRP, C-reactive protein.

* Sample size for ‘excess body weight’ (N = 4,942) and ‘obesity’ (N = 2,753) categories.

* Excess body weight was denoted by combining participants in the ‘overweight’ and ‘obesity’ categories.

*Excess body weight.* CRP acted as a partial mediator of the association between excess body weight (overweight + obesity) and elevated somatic symptoms, explaining a total of 14.92% (indirect effect: β = 0.004, 95% CI: 0.001, 0.006). No total (β = 0.023, 95% CI: −0.000, 0.047) or direct (β = 0.020, 95% CI: −0.004, 0.044) effect was found in this model.

*Obesity.* There was no indirect effect of CRP on the association between obesity (versus normal weight) and somatic symptoms (indirect effect: β = 0.004, 95% CI: −0.000, 0.010). However, obesity was associated with somatic symptoms (total effect: β = 0.045, 95% CI: 0.016, 0.077; direct effect: β = 0.041, 95% CI: 0.011, 0.073).

### 3.6 Sensitivity analyses

The results of the first sensitivity analysis examining the associations of BMI categories with continuous overall and domain-specific scores were broadly consistent with those of the main analysis (Supplementary Table S4). The associations of overweight, obesity, and excess body weight (overweight + obesity) with somatic symptoms (overweight: Model 4: B = 0.047, SE = 0.030, 95% CI: −0.011, 0.106; obesity: Model 4: B = 0.174, SE = 0.033, 95% CI: 0.109, 0.240; excess body weight: Model 4: B = 0.095, SE = 0.028, 95% CI: 0.040, 0.149) were stronger than those observed for cognitive-affective symptoms (overweight: Model 4: B = −0.037, SE = 0.038, 95% CI: −0.112, 0.038; obesity: Model 4: B = 0.006, SE = 0.043, 95% CI: −0.078, 0.089; excess body weight: Model 4: B = −0.021, SE = 0.036, 95% CI: −0.091, 0.049). The results also extended the main findings by showing that obesity was related to both somatic (Model 4: B = 0.174, SE = 0.033, 95% CI: 0.109, 0.240) and overall symptoms (Model 4: B = 0.177, SE = 0.064, 95% CI: 0.051, 0.303).

The second sensitivity analysis showed that, after additionally adjusting for antidepressant and anti-inflammatory drugs at wave 6, CRP remained a statistical mediator of the prospective association between excess body weight (overweight + obesity) and elevated somatic symptoms, explaining a total of 15.16% (indirect effect: β = 0.003, 95% CI: 0.001, 0.006) (Supplementary Table S5 and Fig. S1). No total (β = 0.018, 95% CI: −0.004, 0.041) or direct (β = 0.016, 95% CI: −0.008, 0.040) effect was found in this model.

The third sensitivity analysis indicated that compared with the BMI category ‘normal weight + overweight’, there was an indirect effect of CRP on the association between obesity and elevated somatic symptoms (indirect effect: β = 0.003, 95% CI: 0.001, 0.007) (Supplementary Table S6 and Fig. S2). Obesity (versus normal weight + overweight) was associated with somatic symptoms (total effect: β = 0.048, 95% CI: 0.025, 0.073; direct effect: β = 0.045, 95% CI: 0.022, 0.070).

Lastly, in multivariable-adjusted, sex-stratified analyses, the prospective associations of overweight, obesity, and excess body weight with elevated overall, cognitive-affective, and somatic depressive symptoms (path c) were stronger in women compared with men (Supplementary Table S7).

## 4 Discussion

This is the first study to examine the mediating role of systemic low-grade inflammation in the associations of excess body weight with elevated overall, cognitive-affective, and somatic depressive symptoms in a sample of English community-dwelling older adults. Excess body weight (denoted by the combined weight group: overweight + obesity) was associated with elevated somatic, but not cognitive-affective or overall depressive symptoms at a ten-year follow-up. A positive association was also observed for the relationship between CRP and subsequent somatic, but not cognitive-affective or overall depressive symptoms. Mediation analysis revealed that CRP acted as a partial mediator of the association between excess body weight and somatic depressive symptoms, explaining a total of 14.92% of this association.

In the present study, the association of excess body weight with subsequent overall depressive symptoms was weak. These findings somewhat contradict the results of a previous investigation reporting a stronger positive association between excess body weight and overall depression in older adults (Marijnissen et al., 2011). Discrepancies in findings may be ascribed to methodological differences (*e.g.,* operationalisation of constructs). For example, Marjinissen et al. (2011) used continuous scores to measure overall depressive symptoms. Interestingly, our first sensitivity analysis showed that obesity was related to both somatic and overall symptoms when using continuous scores, but not to cognitive-affective symptoms. Our supplementary analysis also revealed stronger associations of overweight/obesity/excess body weight with subsequent depressive symptoms in women compared with men. These results demonstrate that sex might be an effect modifier, emphasising the value of considering sex differences in future studies.

The finding that excess body weight (overweight + obesity), particularly obesity, is associated primarily with elevated somatic symptoms at follow-up is consistent with our second hypothesis and coincides with previous findings in this field of research (Baldofski et al., 2019, Wiltink et al., 2013). One possible explanation for domain-specific effects of obesity is that somatic symptoms may be primarily attributable to subclinical somatic conditions accompanied by obesity, such as altered appetite and sleep problems, which may subsequently induce sickness behaviour and reinforce somatic symptoms of depression (Lamers et al., 2018). Notably, previous evidence suggests that increased appetite is a key factor that drives associations of depression with BMI and CRP (Lamers et al., 2018). Furthermore, a recent genome-wide association study of >25,000 individuals reported that depressed patients with increased appetite and/or weight carried genetic risk variants for both elevated CRP levels and BMI (Milaneschi et al., 2017). These findings and the results presented in this study suggest that excess body weight is associated with a distinct set of depressive symptoms.

We also found evidence for an association of overweight, obesity, and excess body weight (overweight + obesity) with increased levels of CRP at wave 6, independently of baseline CRP, sociodemographic, lifestyle, and illness-related factors. This supports earlier findings showing that in individuals with obesity, adipose tissue dysfunction is a key factor contributing to low-grade inflammation, as indicated by elevated concentrations of CRP and IL-6 (Yudkin et al., 1999). Moreover, the present analysis revealed that higher CRP was associated with subsequently elevated somatic, but not cognitive-affective or overall depressive symptoms ten years later, which provides additional support for the sickness behaviour theory (Dantzer et al., 2008). These results also concur with those reported by White et al. (2017), showing an association between elevated CRP levels and somatic, rather than cognitive-affective symptoms, after controlling for sociodemographic variables, health conditions, and medication use.

Finally, the mediation analysis revealed that CRP acted as a partial mediator of the relationship between excess body weight and somatic symptoms, accounting for a total of 14.92% of this association. This finding is in line with previous evidence supporting the role of obesity-related inflammation in comorbid depression (Castanon et al., 2014). Furthermore, it suggests that adipose tissue promotes pro-inflammatory cytokine secretion and stimulates CRP synthesis (Penninx et al., 2013), which may then activate brain inflammatory processes implicated in depression aetiology (Miller and Raison, 2016). However, although the indirect effect remained following additional adjustment for medication use, it was relatively small in magnitude. This might be due to the fact that CRP cannot directly affect emotion-regulating brain regions as it is not capable of crossing the blood–brain barrier (BBB) (Miller and Raison, 2016). Another explanation is that combining overweight and obesity into a single weight group may have introduced bias. Overweight was not associated with subsequent somatic symptoms in multivariable-adjusted analysis, and only weakly associated with CRP at follow-up. This indicates that obesity is likely to be the driver of the association between excess body weight, CRP, and somatic depressive symptoms. Accordingly, the third sensitivity analysis that compared obesity with ‘normal weight + overweight’ provided further evidence for a mediating role of CRP in the association between obesity and somatic depressive symptoms. Larger prospective cohort studies are needed to replicate our analyses.

The present study has several strengths. The longitudinal design allowed for the assessment of temporality. We used data from a large population-based observational study of older adults living in England. We were able to account for several potential confounders in the analyses. Furthermore, a symptom-focused approach was employed to disentangle potential symptom-specific associations of excess body weight with depression. However, the results of this study should be interpreted in the context of the study’s limitations. First, causal inference is not possible given the observational nature of the study. Although our results support a mediating role of systemic low-grade inflammation in the association between excess body weight and somatic depressive symptoms, CRP is not capable of crossing the blood–brain barrier and cannot directly affect depression-related brain regions. Hence, the use of CRP may not precisely capture the causal pathway by which systemic inflammation affects depression-related brain functioning. Future research may therefore benefit from using other markers of inflammation, such as IL-6, a potent stimulator of CRP synthesis, which was, however, not available in ELSA. Second, the assessment of depressive symptom domains was based on a single self-report measure (CES-D), and the risk of self-report bias cannot be excluded. Third, the somatic symptom domain had low internal consistency, although the categorisation of depressive symptoms into subgroups was informed by recent factor-analytic studies of the 8-item CES-D (Frank et al., 2020, Iob et al., 2020). Fourth, a measure of increased appetite, which has been identified as a key factor driving the association of depression with BMI and CRP (Lamers et al., 2018), was not available in ELSA. Fifth, the ELSA dataset does not include information on other, potentially important confounding factors, such as menopause, which has been found to increase the risk of both obesity and depressive symptoms in women (Bromberger et al., 2010, Davis et al., 2012). Additionally, ELSA does not feature information on specific dietary patterns, such as Mediterranean diets, which have been linked to weight loss (Esposito et al., 2011), reduced levels of circulating inflammatory markers (Luciano et al., 2012), and a lower risk of depression (Lassale et al., 2019). No data were available on chronic liver disease, a systemic inflammatory disease (D'Mello et al., 2009) which has been associated with increased risk of depression (Gutteling et al., 2006). Factors such as obesity and excessive alcohol intake may also affect liver function (Angulo et al., 1999, Savolainen et al., 1993), leading to increased levels of circulating CRP (Yoneda et al., 2007). Furthermore, it has been reported that hepatic inflammation triggers the production of CC-chemokine ligand 2/monocyte chemoattractant protein-1 and monocyte infiltration of the brain, resulting in depressive-like behaviours in rodents (D'Mello et al., 2009), with potential extrapolations to humans. Sixth, the generalisability of results is limited to white, older adults. Lastly, relative to the analytical sample, excluded participants were more likely to smoke and to have a lower socioeconomic status. Thus, the study results may not be generalisable to this subpopulation.

Our results may have important implications for future research. The finding that CRP acted as a partial mediator of the prospective relationship between excess body weight and somatic symptoms, but not cognitive-affective or overall symptoms, highlights the importance of considering symptom-specific effects in future research. Furthermore, future research is needed to investigate whether targeting low-grade inflammatory processes in adults with excess body weight and somatic depressive symptoms would improve treatment outcomes. Notably, weight loss interventions, such as bariatric surgery have shown efficacy in lowering both levels of depressive symptoms and inflammatory markers in individuals with obesity (Emery et al., 2007). Future clinical trials should investigate whether these effects are associated with a reduction in all or just specific depressive symptoms.

## 5 Conclusions

In conclusion, the present study provides evidence for a mediating role of CRP in the association of excess body weight with elevated somatic, but not overall or cognitive-affective depressive symptoms in older adults. Although the mediating effect of CRP was modest, our findings highlight the importance of considering inflammatory mechanisms in the link between excess body weight and specific depressive symtoms in future research. The exploration of symptom-specific associations may help inform the design of more tailored interventions for individuals with distinct risk factor-based symptom profiles.

## Ethical approval

Ethical approval for each of the ELSA waves was granted from the National Research Ethics Service (London Multi-Centre Research Ethics Committee) (MREC/01/2/91) (https://www.nres.npsa.nhs.uk). All participants provided informed consent.

## Funding

The English Longitudinal Study of Ageing (ELSA) is funded by the National Institute on Ageing (R01AG017644) and by a consortium of UK government departments coordinated by the Economic and Social Research Council (ESRC).

Dorina Cadar is supported by the National Institute on Ageing (R01AG017644) and the ESRC (ES/T012091/1, & ES/S013830/1).

Philipp Frank is supported by the ESRC and the Biotechnology and Biological Sciences Research Council (BBSRC) (ES/P000347/1).

Eleonora Iob is supported by a Wellcome Trust Sir Henry Wellcome fellowship (222750/Z/21/Z, 2021-2025).

## Contributors

Keqin Chu, Dr Dorina Cadar, and Philipp Frank proposed, designed, and conducted this study. Data were extracted by Dr Dorina Cadar and Philipp Frank. Dr Dorina Cadar, Philipp Frank, and Dr Eleonora Iob proposed the analytic plan and provided support on software use. Analysis and interpretation of the data were carried out by Keqin Chu, with support from Dr Dorina Cadar, Dr Eleonora Iob, and Philipp Frank. The project write-up was completed by Keqin Chu. All authors reviewed the draft manuscript.

Dr Dorina Cadar, Keqin Chu, and Philipp Frank had full access to all the data in the study. Keqin Chu conducted the data analysis and takes responsibility for the integrity of the data and accuracy of the data analysis.

## Availability of data and materials

The ELSA was developed by a team of researchers based at University College London, the Institute for Fiscal Studies, and the National Centre for Social Research.

The data are linked to the UK Data Archive and freely available through the UK data services and can be accessed here: https://discover.ukdataservice.ac.uk.

## Declaration of Competing Interest

The authors declare that they have no known competing financial interests or personal relationships that could have appeared to influence the work reported in this paper.

## Data Availability

Data will be made available on request.
