# Supplementary material for: Excess body weight and specific types of depressive symptoms: Is there a mediating role of systemic low-grade inflammation?
Source: Brain Behav Immun. 2023 Feb;108:233–44. doi: 10.1016/j.bbi.2022.11.016 (PMC10567582; doi:10.1016/j.bbi.2022.11.016)
Supplement: Supplementary data 1 [file mmc1.docx]

**Supplementary material**

**Power analysis**

A post-hoc power-analysis was conducted (alpha level 0.05) in G*Power 3.1 (Faul et al., 2009) to estimate the minimum sample size needed to achieve a power of 0.80. The expected R-squared between the predictors and over covariates was set at a moderate level of 0.25 (Cohen, 2013). The event rates under H_0_ and H_1_ were set at 0.30 and 0.40, respectively (Vollmer et al., 2015). The results indicated that a minimum sample size of 954 participants was required for the present study to achieve a 0.80 power (alpha level 0.05).

**References**

Cohen, J. (2013). *Statistical Power Analysis for the Behavioral Sciences*. Routledge. <https://doi.org/10.4324/9780203771587>

Faul, F., Erdfelder, E., Buchner, A., & Lang, A. G. (2009). Statistical power analyses using G* Power 3.1: Tests for correlation and regression analyses. *Behavior Research Methods*, *41*(4), 1149–1160. <https://doi.org/10.3758/BRM.41.4.1149>

Vollmer, R. L., Adamsons, K., Gorin, A., Foster, J. S., & Mobley, A. R. (2015). Investigating the relationship of body mass index, diet quality, and physical activity level between fathers and their preschool-aged children. *Journal of the Academy of Nutrition and Dietetics*, *115*(6), 919–926. <https://doi.org/10.1016/j.jand.2014.12.003>

K. Chu et al. ELSA_BMI_depressive symptoms_inflammation

| Supplementary Table S1. Descriptive characteristics of the analytic sample before and after multiple imputations. | | | |
| --- | --- | --- | --- |
|  | **Observed (N = 5,274)** | **Missing** | **Imputed** |
|  | **Mean (SD) / Nr. (%)** | **% (Counts)** | **Mean (SD) / Nr. (%)** |
| Elevated overall depressive symptoms (CES-D ≥ 4)  wave 9  wave 4 | 412 (10.87%)  633 (12.14%) | 28.14 (1,484)  1.10 (58) | 711 (13.48%)  643 (12.19%) |
| Elevated cognitive-affective symptoms (upper tertile: CES-D ≥ 2)  wave 9  wave 4 | 997 (26.28%)  776 (14.87%) | 31.92 (1,683)  1.04 (55) | 833 (15.79%)  793 (15.04%) |
| Elevated somatic symptoms (upper tertile: CES-D ≥ 2)  wave 9  wave 4 | 721 (18.92%)  856 (16.35%) | 27.76 (1,464)  0.70 (37) | 1,182 (22.41%)  861 (16.33%) |
| BMI at wave 4 (kg/m^2^)  *Underweight (BMI < 18.5)*  *Normal weight (18.5 ≤ BMI < 25)*  *Overweight (25 ≤ BMI < 30)*  *Obesity (BMI ≥ 30)* | 32 (0.68%)  1,250 (26.45%)  2,052 (43.42%)  1,392 (29.45%) | 9.80 (517) | 34 (0.64%)  1,381 (26.19%)  2,289 (43.40%)  1,570 (29.77%) |
| C-reactive protein at wave 6 (mg/L)*  wave 6  wave 4 | 0.80 (0.8, 3.3)  0.86 (0.8, 3.3) | 0.00 (0)  21.79 (1,149) | 0.70 (0.8, 3.3)  0.87 (0.9, 3.8) |
| Age | 64.14 ± 8.57 | 0.00 (0) | 64.14 ± 8.57 |
| Sex  *Men*  *Women* | 2,371 (44.96%)  2,903 (55.04%) | 0.00 (0) | 2,371 (44.96%)  2,903 (55.04%) |
| Education  *University degree*  *Less than university*  *No qualification* | 1,930 (37.50%)  2,047 (39.78%)  1,169 (22.72%) | 2.43 (128) | 1,988 (37.69%)  2,100 (39.82%)  1,186 (22.49%) |
| Wealth (quintiles)*  *Lowest quintile*  *2nd quintile*  *3rd quintile*  *4th quintile*  *Highest quintile* | 688 (13.05%)  949 (17.99%)  1,024 (19.42%)  1,095 (20.76%)  1,245 (23.61%) | 5.18 (273) | 711 (13.48%)  986 (18.70%)  1,081 (20.50%)  1,168 (22.15%)  1,328 (25.18%) |
| Marital status  *Single*  *Married* | 1,590 (30.15%)  3,684 (69.85%) | 0.00 (0) | 1,590 (30.15%)  3,684 (69.85%) |
| Smoking status  *Smoker*  *Non-smoker* | 664 (12.59%)  4,573 (86.71%) | 0.70 (37) | 665 (12.61%)  4,609 (87.39%) |
| Alcohol consumption  *Less than daily*  *Daily (5/7 per week)* | 3,631 (68.85%)  1,122 (21.27%) | 9.88 (521) | 4,044 (76.68%)  1,230 (23.32%) |
| Sedentary lifestyle  *Yes*  *No* | 609 (11.55%)  4,664 (88.43%) | 0.02 (1) | 610 (11.57%)  4,664 (88.43%) |
| Coronary heart disease ever  *Yes*  *No* | 334 (6.33%)  4,940 (93.67%) | 0.00 (0) | 334 (6.33%)  4,940 (93.67%) |
| Stroke ever  *Yes*  *No* | 139 (2.64%)  5,135 (97.36%) | 0.00 (0) | 139 (2.64%)  5,135 (97.36%) |
| Diabetes ever  *Yes*  *No* | 404 (7.66%)  4,870 (92.34%) | 0.00 (0) | 404 (7.66%)  4,870 (92.34%) |
| Cancer ever  *Yes*  *No* | 375 (7.11%)  4,899 (92.89%) | 0.00 (0) | 375 (7.11%)  4,899 (92.89%) |
| Antidepressant drugs at wave 6  *Yes*  *No* | 555 (10.52%)  4,719 (89.48%) | 0.00 (0) | 555 (10.52%)  4,719 (89.48%) |
| Anti-inflammatory drugs at wave 6  *Yes*  *No* | 418 (7.93%)  4,856 (92.07%) | 0.00 (0) | 418 (7.93%)  4,856 (92.07%) |
| *Note:* Mean (standard deviation) (95% confidence intervals), and Number (percentage).  Abbreviations: BMI, body mass index; CES-D, 8-item Centre for Epidemiological Studies Depression.  * C-reactive protein was based on the geometric mean and interquartile ranges.  * The cut-off points for the wealth group definition were: Lowest – less than £60k; 2^nd^ – between £60k-£201k; 3^rd^ – between £201k-£303k; 4^th^ – between £303k-£496k; Highest – more than £496k. | | | |

K. Chu et al. ELSA_BMI_depressive symptoms_inflammation

| Supplementary Table S2. Baseline C-reactive protein of the analytic sample stratified by weight and depression status. | | | | | | | |
| --- | --- | --- | --- | --- | --- | --- | --- |
| **Total sample (N = 4,942)** | **Weight (kg/m^2^)** | | | ***P-value*** | **Depression status** | | ***P-value*** |
|  | Normal weight  (18.5 ≤ BMI < 25)  (N = 1,318) | Overweight  (25 ≤ BMI < 30)  (N = 2,189) | Obesity  (BMI ≥ 30)  (N = 1,435) |  | Non-elevated  (CES-D < 4)  (N = 4,365) | Elevated  (CES-D ≥ 4)  (N = 577) |  |
| C-reactive protein (mg/L),  mean (SD)* | 0.13 ± 1.07 | 0.56 ± 1.01 | 1.00 ± 0.95 | <0.001 | 0.54 ± 1.05 | 0.84 ± 1.11 | <0.001 |
| C-reactive protein (mg/L),  N (%)  CRP ≤ 3  3 < CRP < 10  CRP ≥ 10 | 1,084 (82.25%)  200 (15.17%)  34 (2.58%) | 1,594 (72.82%)  498 (22.75%)  97 (4.43%) | 788 (54.91%)  549 (38.26%)  98 (6.83%) | <0.001 | 3,126 (71.62%)  1,055 (24.17%)  184 (4.22%) | 340 (58.93%)  192 (33.28%)  45 (7.80%) | <0.001 |
| *Note:* Mean (standard deviation) and Number (percentage).  Abbreviations: BMI, body mass index; CRP, C-reactive protein; CES-D, 8-item Centre for Epidemiological Studies Depression.  * C-reactive protein was based on the arithmetic mean of Log C-reactive protein. | | | | | | | |

K. Chu et al. ELSA_BMI_depressive symptoms_inflammation

| Supplementary Table S3. Comparison of sample characteristics between included and excluded participants at baseline (wave 4, 2008/09). | | | | | |
| --- | --- | --- | --- | --- | --- |
|  | **Included participants**  **(N = 4,942)** | | **Excluded participants**  **(N = 6,108)** | | **Group comparisons** |
|  | **N** | **Mean (SD) / Nr. (%)** | **N** | **Mean (SD) / Nr. (%)** | ***p-value**** |
| BMI at wave 4 (kg/m^2^)  *Underweight (BMI < 18.5)*  *Normal weight (18.5 ≤ BMI < 25)*  *Overweight (25 ≤ BMI < 30)*  *Obesity (BMI ≥ 30)* | 4,942 | 1,318 (26.67%)  2,189 (44.29%)  1,435 (29.04%) | 3,868 | 73 (1.89%)  967 (25.00%)  1,500 (38.78%)  1,328 (34.33%) | < 0.001 |
| Age | 4,942 | 64.00 ± 8.51 | 6,108 | 66.25 ± 11.86 | 0.112 |
| Sex  *Men*  *Women* | 4,942 | 2,232 (45.16%)  2,710 (54.84%) | 6,108 | 2,693 (44.09%)  3,415 (55.91%) | 0.687 |
| Education  *University degree*  *Less than university*  *No qualification* | 4,942 | 1,900 (38.45%)  1,957 (39.60%)  1,085 (21.95%) | 5,867 | 1,734 (29.56%)  2,222 (37.87%)  1,911 (32.57%) | < 0.001 |
| Wealth (quintiles)*  *Lowest quintile*  *2nd quintile*  *3rd quintile*  *4th quintile*  *Highest quintile* | 4,942 | 634 (12.83%)  903 (18.27%)  1,017 (20.58%)  1,108 (22.42%)  1,280 (25.90%) | 4,920 | 1,107 (22.50%)  1,017 (20.67%)  957 (19.45%)  961 (19.53%)  878 (17.85%) | < 0.001 |
| Marital status  *Single*  *Married* | 4,942 | 1,445 (29.24%)  3,497 (70.76%) | 6,106 | 2,093 (34.28%)  4,013 (65.72%) | 0.056 |
| Smoking status  *Smoker*  *Non-smoker* | 4,942 | 597 (12.08%)  4,345 (87.92%) | 6,038 | 941 (15.58%)  5,097 (84.42%) | < 0.001 |
| Alcohol consumption  *Less than daily*  *Daily (5/7 per week)* | 4,942 | 3,788 (76.65%)  1,154 (23.35%) | 4,663 | 3,611 (77.44%)  1,052 (22.56%) | < 0.001 |
| Sedentary lifestyle  *Yes*  *No* | 4,942 | 535 (10.83%)  4,407 (89.17%) | 6,066 | 1,457 (24.02%)  4,609 (75.98%) | 0.056 |
| Coronary heart disease ever  *Yes*  *No* | 4,942 | 302 (6.11%)  4,640 (93.89%) | 6,108 | 669 (10.95%)  5,439 (89.05%) | 0.379 |
| Stroke ever  *Yes*  *No* | 4,942 | 128 (2.59%)  4,814 (97.41%) | 6,108 | 371 (6.07%)  5,737 (93.93%) | 0.784 |
| Diabetes ever  *Yes*  *No* | 4,942 | 362 (7.32%)  4,580 (92.68%) | 6,108 | 733 (12.00%)  5,375 (88.00%) | 0.224 |
| Cancer ever  *Yes*  *No* | 4,942 | 349 (7.06%)  4,593 (92.94%) | 6,108 | 539 (8.82%)  5,569 (91.18%) | 0.856 |
| Antidepressant drugs at wave 6  *Yes*  *No* | 4,942 | 498 (10.08%)  4,444 (89.92%) | 2,052 | 270 (13.16%)  1,782 (86.84%) | 0.161 |
| Anti-inflammatory drugs at wave 6  *Yes*  *No* | 4,942 | 373 (7.55%)  4,569 (92.45%) | 2,052 | 150 (7.31%)  1,902 (92.69%) | 0.177 |
| *Note:* Mean (standard deviation) (95% confidence intervals), and Number (percentage).  Abbreviations: BMI, body mass index; CES-D, 8-item Centre for Epidemiological Studies Depression.  * p-value estimates from tests including t-tests (continuous variables) and chi-square tests (categorical variables).  * The cut-off points for the wealth group definition were: Lowest – less than £60k; 2^nd^ – between £60k-£201k; 3^rd^ – between £201k-£303k; 4^th^ – between £303k-£496k; Highest – more than £496k. | | | | | |

K. Chu et al. ELSA_BMI_depressive symptoms_inflammation

| Supplementary Table S4. Linear regression analysis of baseline excess body weight, overweight, and obesity (versus normal weight) (wave 4, 2008/09) with overall, cognitive-affective, and somatic depressive symptoms at follow-up (wave 9, 2018/19). | | | | | | | | | |
| --- | --- | --- | --- | --- | --- | --- | --- | --- | --- |
| **Model** | **Overall symptoms** | | | **Cognitive-affective symptoms** | | | **Somatic symptoms** | | |
|  | **B (SE)** | **95% CI** | ***P-value*** | **B (SE)** | **95% CI** | ***P-value*** | **B (SE)** | **95% CI** | ***P-value*** |
| *Excess body weight**  (N = 4,942)  Model 1  Model 2  Model 3  Model 4 | 0.102 (0.054)  0.067 (0.053)  0.074 (0.054)  0.072 (0.054) | -0.003, 0.207  -0.038, 0.172  -0.031, 0.179  -0.033, 0.178 | *0.056*  *0.211*  *0.167*  *0.179* | -0.001 (0.035)  -0.024 (0.035)  -0.018 (0.035)  -0.021 (0.036) | -0.070, 0.068  -0.094, 0.045  -0.087, 0.052  -0.091, 0.049 | *0.977*  *0.491*  *0.622*  *0.554* | 0.107 (0.028)  0.093 (0.028)  0.094 (0.028)  0.095 (0.028) | 0.052, 0.161  0.039, 0.147  0.039, 0.148  0.040, 0.149 | *<0.001*  *0.001*  *0.001*  *0.001* |
| *Overweight*  (N = 3,507)  Model 1  Model 2  Model 3  Model 4 | 0.007 (0.058)  -0.003 (0.058)  0.011 (0.058)  0.009 (0.058) | -0.107, 0.121  -0.117, 0.110  -0.103, 0.124  -0.104, 0.122 | *0.906*  *0.954*  *0.854*  *0.874* | -0.036 (0.038)  -0.044 (0.038)  -0.035 (0.038)  -0.037 (0.038) | -0.111, 0.039  -0.119, 0.030  -0.110, 0.040  -0.112, 0.038 | *0.349*  *0.245*  *0.354*  *0.332* | 0.043 (0.030)  0.042 (0.030)  0.047 (0.030)  0.047 (0.030) | -0.016, 0.102  -0.017, 0.100  -0.011, 0.106  -0.011, 0.106 | *0.150*  *0.165*  *0.114*  *0.114* |
| *Obesity*  (N = 2,753)  Model 1  Model 2  Model 3  Model 4 | 0.245 (0.063)  0.177 (0.064)  0.177 (0.064)  0.177 (0.064) | 0.121, 0.369  0.053, 0.302  0.052, 0.303  0.051, 0.303 | *<0.001*  *0.005*  *0.006*  *0.006* | 0.051 (0.042)  0.007 (0.042)  0.011 (0.042)  0.006 (0.043) | -0.031, 0.132  -0.075, 0.089  -0.071, 0.094  -0.078, 0.089 | *0.225*  *0.866*  *0.786*  *0.896* | 0.203 (0.033)  0.174 (0.033)  0.169 (0.033)  0.174 (0.033) | 0.138, 0.267  0.110, 0.239  0.104, 0.234  0.109, 0.240 | *<0.001*  *<0.001*  *<0.001*  *<0.001* |
| *Note:* Model 1: Effect estimates adjusted for age, sex, and baseline depressive symptoms. Model 2: model 1 + education, wealth, and marital status. Model 3: model 2 + smoking status, alcohol consumption, and sedentary lifestyle. Model 4: model 3 + coronary heart disease, stroke, diabetes, and cancer.  Abbreviations: B, regression coefficient; CI, confidence intervals; SE, Standard error.  * Excess body weight was denoted by combining participants in the ‘overweight’ and ‘obesity’ categories. | | | | | | | | | |

K. Chu et al. ELSA_BMI_depressive symptoms_inflammation

| Supplementary Table S5. Mediation of the associations of baseline excess body weight and obesity (versus normal weight) (wave 4, 2008/09) with somatic depressive symptoms at wave 9 (2018/19) through C-reactive protein at wave 6 (2012/13), additionally controlling for antidepressant and anti-inflammatory drugs (wave 6, 2012/13) (N = 4,942; N = 2,753)*. | | | | | | |
| --- | --- | --- | --- | --- | --- | --- |
| **Independent variable (wave 4)** | **Mediator (wave 6)** | **Outcome variable**  **(wave 9)** | **Total indirect effect**  **(a*b)** | **Total effect**  **(c)** | **Total direct effect**  **(c’)** | **Total effect mediated** |
|  |  |  | **Coefficient (Bc CI)** | **Coefficient (Bc CI)** | **Coefficient (Bc CI)** | **%** |
| Excess body weight* | CRP | Somatic symptoms | 0.003 (0.001, 0.006) | 0.018 (-0.004, 0.041) | 0.016 (-0.008, 0.040) | 15.16% |
| Obesity | CRP | Somatic symptoms | 0.004 (-0.001, 0.009) | 0.039 (0.010, 0.069) | 0.035 (0.005, 0.066) |  |
| *Note:* Effect estimates adjusted for sociodemographic variables (age, sex, education, wealth, and marital status), behavioural factors (smoking status, alcohol consumption, and sedentary lifestyle), chronic conditions (coronary heart disease, stroke, diabetes, and cancer), in addition to baseline C-reactive protein and depressive symptoms.  Abbreviations: Bc CI, Bias corrected 95% confidence intervals; CRP, C-reactive protein.  * Sample size for ‘excess body weight’ (N = 4,942) and ‘obesity’ (N = 2,753) categories.  * Excess body weight was denoted by combining participants in the ‘overweight’ and ‘obesity’ categories. | | | | | | |

K. Chu et al. ELSA_BMI_depressive symptoms_inflammation

| Supplementary Table S6. Mediation of the association of obesity (versus normal weight + overweight) (wave 4, 2008/09) with somatic depressive symptoms at wave 9 (2018/19) through C-reactive protein at wave 6 (2012/13) (N = 4,942). | | | | | | |
| --- | --- | --- | --- | --- | --- | --- |
| **Independent variable**  **(wave 4)** | **Mediator**  **(wave 6)** | **Outcome variable**  **(wave 9)** | **Total indirect effect (a*b)** | **Total effect**  **(c)** | **Total direct effect**  **(c’)** | **Total effect mediated** |
|  |  |  | **Coefficient (Bc CI)** | **Coefficient (Bc CI)** | **Coefficient (Bc CI)** | **%** |
| Obesity vs. normal weight + overweight | CRP | Somatic symptoms | 0.003 (0.001, 0.007) | 0.048 (0.025, 0.073) | 0.045 (0.022, 0.070) | 7.26% |
| *Note:* Effect estimates adjusted for sociodemographic variables (age, sex, education, wealth, and marital status), behavioural factors (smoking status, alcohol consumption, and sedentary lifestyle), chronic conditions (coronary heart disease, stroke, diabetes, and cancer), in addition to baseline C-reactive protein and depressive symptoms.  Abbreviations: Bc CI, Bias corrected 95% confidence intervals; CRP, C-reactive protein. | | | | | | |

K. Chu et al. ELSA_BMI_depressive symptoms_inflammation

| Supplementary Table S7. Associations of baseline excess body weight, overweight, and obesity (versus normal weight) (wave 4, 2008/09) with overall, cognitive-affective, and somatic depressive symptoms at follow-up (wave 9, 2018/19) stratified by sex. | | | | | | |
| --- | --- | --- | --- | --- | --- | --- |
| **Total sample**  **(N = 4,942)** | **Males**  **(N = 2,232)** | | | **Females**  **(N = 2,710)** | | |
| **Model** | **Elevated overall symptoms**  **(CES-D ≥ 4)** | **Elevated cognitive-affective symptoms**  **(upper tertile)** | **Elevated somatic symptoms**  **(upper tertile)** | **Elevated overall symptoms**  **(CES-D ≥ 4)** | **Elevated cognitive-affective symptoms**  **(upper tertile)** | **Elevated somatic symptoms**  **(upper tertile)** |
|  | **OR (95% CI)** | **OR (95% CI)** | **OR (95% CI)** | **OR (95% CI)** | **OR (95% CI)** | **OR (95% CI)** |
| *Excess body weight**  (N = 4,942)  Model 1  Model 2  Model 3  Model 4 | 1.032 (0.697, 1.529)  1.015 (0.681, 1.514)  1.019 (0.681, 1.526)  0.991 (0.660, 1.490) | 0.787 (0.559, 1.110)  0.764 (0.539, 1.082)  0.760 (0.535, 1.081)  0.731 (0.512, 1.043) | 1.170 (0.866, 1.579)  1.199 (0.882, 1.629)  1.227 (0.898, 1.675)  1.209 (0.884, 1.655) | 1.204 (0.945, 1.534)  1.106 (0.863, 1.418)  1.156 (0.898, 1.487)  1.162 (0.902, 1.496) | 1.185 (0.951, 1.477)  1.096 (0.874, 1.373)  1.149 (0.914, 1.445)  1.154 (0.917, 1.452) | **1.332** (1.079, 1.646)*  1.237 (0.996, 1.537)  1.236 (0.993, 1.540)  **1.250** (1.003, 1.559)* |
| *Overweight*  (N = 3,507)  Model 1  Model 2  Model 3  Model 4 | 0.911 (0.598, 1.387)  0.927 (0.605, 1.420)  0.947 (0.616, 1.455)  0.918 (0.596, 1.415) | 0.718 (0.496, 1.038)  0.722 (0.497, 1.049)  0.729 (0.501, 1.062)  0.697 (0.477, 1.019) | 0.979 (0.710, 1.349)  1.024 (0.738, 1.420)  1.065 (0.765, 1.484)  1.048 (0.752, 1.463) | 1.040 (0.792, 1.365)  1.007 (0.765, 1.327)  1.053 (0.797, 1.390)  1.063 (0.804, 1.405) | 1.030 (0.804, 1.320)  0.996 (0.775, 1.280)  1.042 (0.809, 1.342)  1.047 (0.813, 1.350) | 1.094 (0.864, 1.386)  1.061 (0.834, 1.349)  1.074 (0.843, 1.368)  1.085 (0.851, 1.384) |
| *Obesity*  (N = 2,753)  Model 1  Model 2  Model 3  Model 4 | 1.260 (0.805, 1.972)  1.173 (0.743, 1.851)  1.152 (0.724, 1.832)  1.132 (0.708, 1.811) | 0.920 (0.615, 1.375)  0.841 (0.558, 1.267)  0.818 (0.539, 1.240)  0.794 (0.521, 1.211) | **1.559** (1.110, 2.191)*  **1.543** (1.089, 2.185)*  **1.547** (1.086, 2.205)*  **1.540** (1.077, 2.203)* | **1.423** (1.081, 1.873)*  1.242 (0.934, 1.651)  1.303 (0.975, 1.742)  1.305 (0.975, 1.748) | **1.396** (1.085, 1.796)*  1.234 (0.951, 1.602)  **1.306** (1.002, 1.704)*  **1.313** (1.006, 1.715)* | **1.678** (1.319, 2.134)***  **1.498** (1.168, 1.921)**  **1.483** (1.152, 1.909)**  **1.506** (1.168, 1.943)** |
| *Note:* Model 1: Effect estimates adjusted for age, sex, and baseline depressive symptoms. Model 2: model 1 + education, wealth, and marital status. Model 3: model 2 + smoking status, alcohol consumption, and sedentary lifestyle. Model 4: model 3 + coronary heart disease, stroke, diabetes, and cancer.  Abbreviations: CES-D, 8-item Centre for Epidemiological Studies Depression; CI, confidence intervals; OR, odds ratio.  Significant associations are shown in bold.  * Excess body weight was denoted by combining participants in the ‘overweight’ and ‘obesity’ categories.  * =p < 0.05, ** =p < 0.005, *** =p < 0.001. | | | | | | |

K. Chu et al. ELSA_BMI_depressive symptoms_inflammation

**
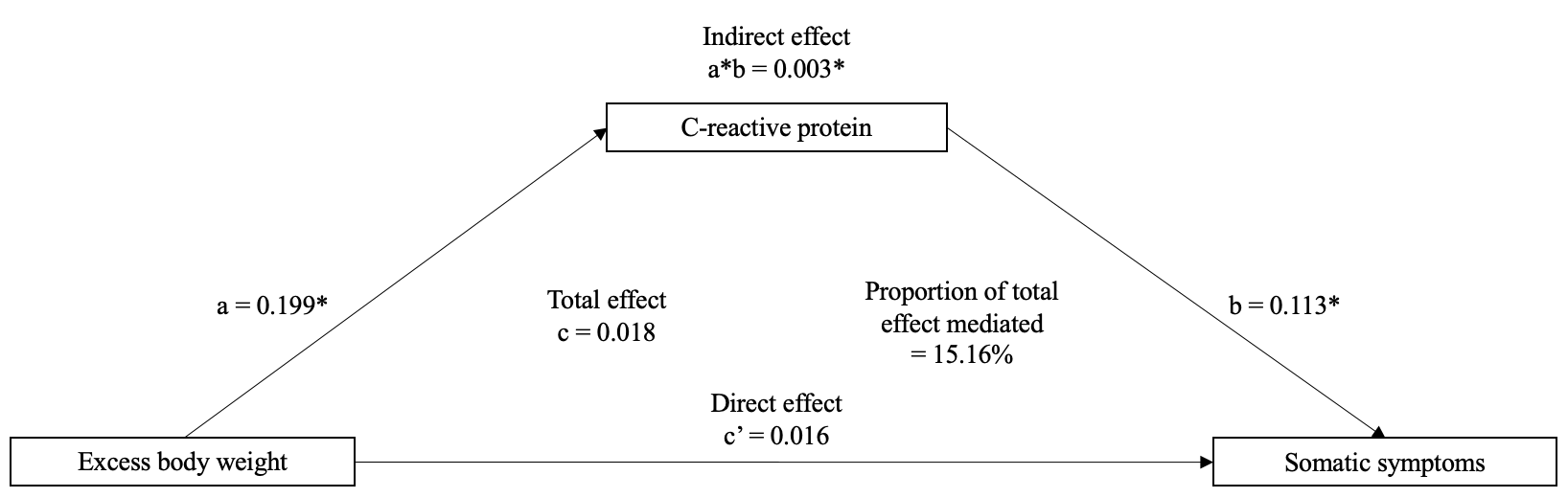
**

**Supplementary Figure S1.** Mediation of the association between excess body weight (denoted by overweight + obesity) (versus normal weight) (wave 4, 2008/09) and subsequent somatic depressive symptoms (wave 9, 2018/19) via C-reactive protein (wave 6, 2012/13), additionally adjusted for antidepressant and anti-inflammatory drugs (wave 6, 2012/13) (N = 4,942).

*Note. * = p < 0.05*

Odds ratios were adjusted for sociodemographic variables (age, sex, education, wealth, and marital status), behavioural factors (smoking status, alcohol consumption, and sedentary lifestyle), chronic conditions (coronary heart disease, stroke, diabetes, and cancer), in addition to baseline C-reactive protein and depressive symptoms. A bias-corrected bootstrap using 1000 iterations was applied to all models.

K. Chu et al. ELSA_BMI_depressive symptoms_inflammation

**
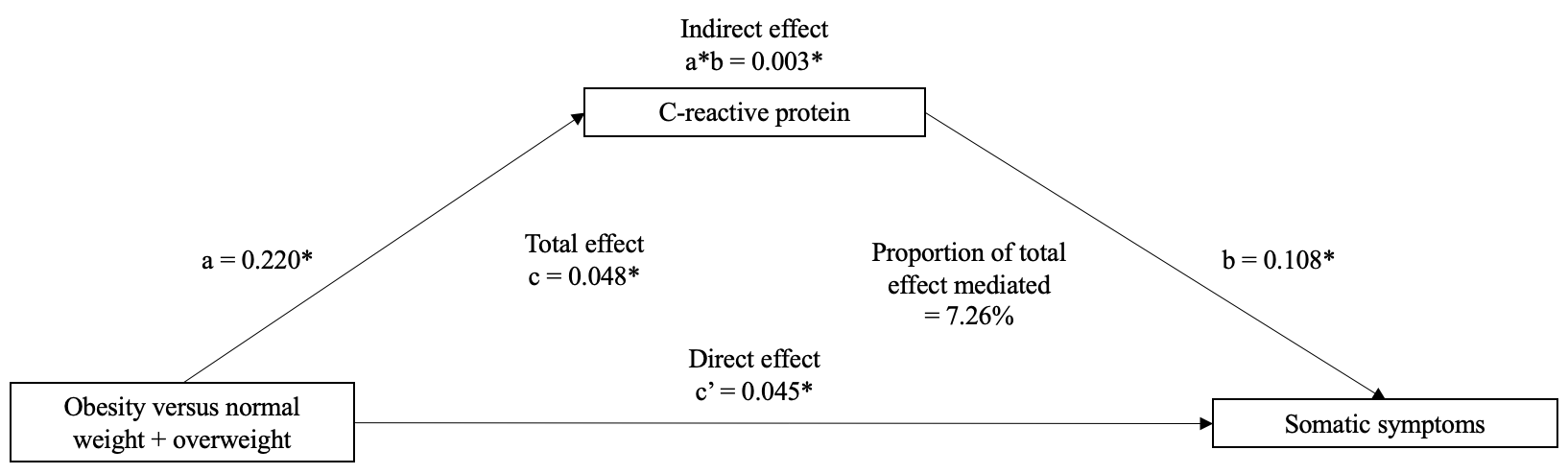
**

**Supplementary Figure S2.** Mediation of the association between obesity (versus normal weight + overweight) (wave 4, 2008/09) and subsequent somatic depressive symptoms (wave 9, 2018/19) via C-reactive protein (wave 6, 2012/13) (N = 4,942).

*Note. * = p < 0.05*

Odds ratios were adjusted for sociodemographic variables (age, sex, education, wealth, and marital status), behavioural factors (smoking status, alcohol consumption, and sedentary lifestyle), chronic conditions (coronary heart disease, stroke, diabetes, and cancer), in addition to baseline C-reactive protein and depressive symptoms. A bias-corrected bootstrap using 1000 iterations was applied to all models.
